# Les facteurs maternels associés au faible poids de naissance: étude cas-témoins dans un hôpital public marocain

**DOI:** 10.11604/pamj.2015.20.303.2659

**Published:** 2015-03-30

**Authors:** Samira Hassoune, Said Bassel, Samira Nani, Hicham Elbouri, Karima Zine, Abderrahmane Maaroufi

**Affiliations:** 1 Laboratoire d'Epidémiologie, Faculté de Médecine et de Pharmacie, Université Hassan II, Casablanca, Maroc

**Keywords:** Faible poids de naissance, facteurs de risque, grossesse, nouveau-né, Mohammedia, low birth weight, risk factor, pregnancy, newborn, Mohammedia

## Aux editeurs du Journal Panafricain de Médecine

Le faible poids de naissance (F.P.N.) est un facteur majeur de morbi-mortalité néonatale et constitue un véritable problème de santé publique dans les pays en développement [1]. C'est un important prédicteur de la survie de l'enfant et de son développement ultérieur [2, 3] puisqu'il prédispose à court et à moyen termeà de nombreuses pathologies telles que le syndrome de détresse respiratoire, les infections, l'entérocolite nécrotique, l'hydrocéphalie et le retard mental [4]. Il augmente également le risque de survenue de certaines affections à l’âge adulte comme la maladie coronarienne, l'hypertension artérielle, le diabète de type 2 et la dépression [5, 6].

Le poids de naissance est un important indicateur de l’état de santé et de la situation nutritionnelle de la mère avant et pendant la grossesse et reflète la qualité des prestations sanitaires [1]. C'est pour cela que nous avons mené une étude cas-témoins dont l'objectif était de déterminer les facteursde risque maternels associés au faible poids de naissance à la province de Mohammedia afin d’élaborer une stratégie de prévention adaptée.

Cette étude s'est déroulée de mai à juillet 2012 à l'hôpital public de Mohammedia. Elle a concerné 30 cas constitués de mères dont les nouveaux nés avaient un faible poids à la naissance et 120 mères témoins dont les nouveaux nés avaient un poids normal. Les grossesses gémellaires ont été exclues de notre étude. Les femmes ont été informées des objectifs de l'enquête et leur consentement a été obtenu avant l'administration du questionnaire.

Les variables maternelles étudiées étaient les données sociodémographiques et économiques, les données anthropométriques, l’âge gestationnel, le suivi prénatal, les pathologies survenues au cours de la grossesse, les facteurs nutritionnels et les habitudes toxiques.

La moyenne d’âge des femmes était de 24,9 (±5,7) ans chez les cas et de 27,4 (±6,6) ans chez les témoins sans différence statistiquement significative (p = 0,06). Parmi les nouveaux nés de FPN, le sexe ratio fille/garçon était 2,75, 7(23,3%) étaient prématurés, 19(23,3%) étaient nés à terme alors que 4 avaient un âge gestationnel non précisé.

Les facteurs sociodémographiques, obstétricaux et ceux liés à l'alimentation et aux habitudes de vie ont été étudié en analyse bivariée (Tableau 1). La fréquence du FPN était plus élevée chez les femmes ayant un niveau d'instruction faible, vivants seules ou dont le revenu familial mensuel était inférieur à 2500 MDHmais la différence n’était pas statistiquement significative. Concernant les facteurs obstétricaux, l'existence d'une morbidité au cours de la grossesse était associée à un FPN du nouveau-né. Les pathologies associées étaientl'infection urinaire (p = 0,047) et les métrorragies au 3ème trimestre de grossesse (p = 0,03). Le nombre de consultations prénatales n’était pas associé au FPN dans notre étude.

**Tableau 1:** Estimation du risque du FPN selon les caractéristiques maternelles à Mohammedia

Facteurs de risque	Cas	Témoins	OR (IC 95%)	p
	n (%)	n (%)	brut	
**Facteurs socio- démographiques:**				
**Age de la mère**				0,08
< 18 ans	2 (6,7)	2 (6,7)	7,85(0,69; 90)	
à 34 ans	26 (86,7)	26 (86,7)	1	
>34 ans	2 (6,7)	2 (6,7)	0,46 (0,1; 2,1)	
**Niveau d'instruction**				0,69
Primaire ou moins	29 (96,7)	110 (91,7)	2,64 (0,32; 21,56)	
Secondaire ou plus	1 (3,3)	10 (8,3)		
**Statut matrimonial**				0,26
Vivant en couple	28 (93,3)	117 (97,5)		
Vivant seule	2 (6,7)	3 (2,5)	2,78 (0,44; 1,73)	
**Revenu familial**				0,07
<2500 MDH	20 (76,9)	64 (57,1)	2,5 (0,93; 6,7)	
≥2500 MDH	6 (23,1)	48 (42,9)		
**Facteurs obstétricaux:**				
**Age gestationnel**				<0,001
< 37 SA	7 (26,9)	1 (0,9)	38,68 (4,5; 332,06)	
≥ 37 SA	19 (73,1)	105 (99,1)		
**Parité**				0,78
Primipare	17 (56,7)	60 (50,4)	1,36 (0,57; 3,25)	
Paucipare (2à3)	10 (33,3)	48 (40,3)		
Multipare(≥ 4)	3 (10,0)	11 (9,2)	1,31 (0,31; 3,58)	
**Intervalle inter génésique**				1,0
<24 mois	1 (7,7)	7 (11,9)	0,61 (0,06; 5,39)	
≥24 mois	12 (92,3)	52 (88,1)		
**Nombre de CPN**				0,41
< 3 CPN	7 (23,3)	19 (15,8)	1,6 (0,54; 4,70)	
≥ 3 CPN	23 (76,7)	101 (84,2)		
**Morbidité au cours de la grossesse**				0,023
Oui	20 (66,7)	50 (41,7)	2,8 (1,21; 6,46)	
Non	10 (33,3)	70 (58,3)		
**Facteurs nutritionnels et habitudes de vie:**				
**Indice de masse corporelle (IMC)**				0,073
Poids normal	21(70,04)	64 (53,3)	0,49 (0,21; 1,16)	
Surpoids	9 (30,0)	56 (46,7)		
**Quantité de l'alimentation**				<0,001
Suffisante	8 (26,7)	90 (75,0)		
Insuffisante	22 (73,3)	30 (25,0)	8,25 (3,33; 20,47)	
**Variété de l'alimentation**				<0,001
Très variée	8 (26,7)	96 (90,0)		
Pas assez variée	22 (73,3)	24 (20,0)	11 (4,36; 27,6)	
**Nombre d'heures de sommeil /j**				0,031
< 8 H	10 (37,0)	20 (16,7)	2,94 (1,118; 7,38)	
≥ 8 H	17 (63,0)	100 (83,3)		
**Tabagisme passif**				0,002
Oui	10 (33,3)	11 (9,2)	4,95 (1,86; 13,2)	
Non	20 (66,7)	109 (90,8)		

Concernant les habitudes de vie, le tabagisme actif et la consommation d'alcool n'ont pas pu être étudiés car ces facteurs étaient absents chez toutes les parturientes. En revanche, le tabagisme passif était significativement associé au FPN. Par ailleurs, les cas avaient plus fréquemment une alimentation insuffisante et peu variée et un nombre d'heures de sommeil par jour plus faible comparés aux témoins.

Afin de contrôler d’éventuels facteurs de confusion, une analyse de régression logistique a été réalisée (Tableau 2). Les facteurs significativement associés étaient l’âge maternel, la prématurité, la variété de l'alimentation et le nombre d'heures de sommeil par jour.

**Tableau 2:** Résultats de l'analyse de régression logistique des facteurs de risque du FPN

Variable	Coefficient β	OR ajusté	IC à 95%	p
Age de la mère	4,21	67,5	1,87 – 2433,6	0,021
Revenu familial	1,64	5,17	0,98 – 27,31	0,053
Prématurité	5,12	167,003	4,45 – 6132,8	0,005
Indice de masse corporelle (IMC)	1,27	3,57	0,76 – 16,73	0,11
Variété de l'alimentation	-2,76	0,063	0,014 – 0,287	<0,001
Nombre d'heures de sommeil ≥ 8 / 24H	-2,6	0,073	0,014 – 0,384	0,002

Ces résultats concordent avec les données d'autres études qui ont conclu que la naissance d'un nouveau-né de FPN était plus fréquente aux extrêmes de la vie reproductive [7] et chez les mères malnutries [8], alors que dormir plus de 8 heures/jour était un facteur protecteur [9]. Nous n'avons pas trouvé d'association avec le nombre de consultations prénatales alors qu'il s'agit d'un facteur reconnu dans la littérature [10]. Ceci pourrait être expliqué par la qualité de la consultation prénatale offerte au niveau du centre de santé qui est souvent réduite à la prise du poids et à la mesure de la tension artérielle.

## Conflits d'intérêts

Les auteurs ne déclarent aucun conflit d'intérêts.

## Contribution des auteurs

Samira Hassoune: élaboration du protocole, rédaction du manuscrit. Said Bassel: collecte des données, révision de l'article; Samira Nani: élaboration du questionnaire, révision de l'article; Hicham Elbouri: analyse des données, révision de l'article; Karima Zine: analyse des données, révision de l'article; Abderrahmane Maaroufi: supervision de l’étude, révision de l'article. Tous les auteurs ont lu et approuvé la version finale de ce manuscrit.
